# The Practice of Pain Assessment and Management in a Tertiary Oncology Center

**DOI:** 10.7759/cureus.18837

**Published:** 2021-10-17

**Authors:** Nabil ALMouaalamy, Ziyad M Alharbi, Faisal M Aldosari, Saif A Saif, Enad F Alsulimani, Mohammed K Aldawsari, Jamilah AlRahimi

**Affiliations:** 1 Oncology Department/Palliative, Princess Noorah Oncology Center, King Abdulaziz Medical City, National Guard Health Affairs, Jeddah, SAU; 2 Research, King Abdullah International Medical Research Center, Jeddah, SAU; 3 College of Medicine, King Saud Bin Abdulaziz University for Health Sciences, Jeddah, SAU; 4 Cardiology, King Abdulaziz Medical City, King Faisal Cardiac Center, Jeddah, SAU

**Keywords:** opioids, palliative, pain assessment, oncology, cancer pain

## Abstract

Background

Pain is one of the common and devastating symptoms that affects millions of cancer patients globally. Despite published guidelines and education on the assessment and management of cancer-related pain, underestimated or undertreated pain continues to be a considerable worldwide public health concern among cancer patients. In this study, we aimed to assess physicians’ adherence to the World Health Organization (WHO) guidelines in the management and assessment of pain in oncology patients based on the available score of pain in the Princess Noorah Oncology Center (PNOC) at the King Abdulaziz Medical City in Jeddah.

Methodology

This cross-sectional, retrospective chart review study studied 451 patients (selected through computerized random sampling) who were admitted to the PNOC during the study period.

Results

The pain was assessed using the Brief Pain Inventory in almost all patients (n = 450, 99.8%). The pain was categorized as mild in 386 (85.6%) patients, moderate in 46 (10.2%) patients, and severe in 19 (4.2%) patients. Opioid prescriptions were significantly higher among patients with moderate (76.1%) and severe pain (89.5%) compared to those with mild pain (39.1%; p < 0.0001).

Conclusions

The practice of pain documentation for cancer patients was adequate as indicated by reporting the pain scores of 99.8% of inpatients. Patients with moderate and severe pain were more likely to receive opioids and a combination of opioids plus non-opioid analgesics, whereas the prescription of analgesics was predicted by experiencing moderate cancer pain.

## Introduction

Pain is one of the common and devastating symptoms that affects millions of cancer patients globally. On average, 30-50% of patients undergoing active cancer therapy and 75-90% with advanced disease suffer from chronic pain that necessitates pain management [1-3]. Pain is an undesirable sensation that has sensory and emotional aspects and has a considerable effect on cancer patients’ quality of life in terms of physical, behavioral, and social wellbeing [2,4,5]. However, among all cancer patients who show symptoms of pain, almost 30-50% suffer from moderate-to-severe pain [6,7]. Although this pain is manageable, it can affect patients’ quality of life negatively [6,7].

Inefficient treatment of cancer pain is a frequently reported issue despite the available effective therapies and pain management guidelines. Moreover, the application of pain management guidelines in clinical practice is still problematic [8]. A review assessing compliance with the Joint Commission standards for pain management in patients with cancer showed that pain intensity and pain reassessment were documented in 53% and 44%, respectively [8]. Moreover, unsuitable pain assessment postpones pain relief [8]. According to the World Health Organization (WHO), the goal of cancer pain management is to relieve pain to a level that allows for an acceptable quality of life [9]. Inadequate pain relief can be due to some barriers such as improper identification, assessment, or documentation of the degree of pain [10]. The WHO recommends starting with a combination of mild analgesics (paracetamol and/or non-steroidal anti-inflammatory drugs [NSAIDs]) with an opioid as initial management of moderate-to-severe pain and patients should not be started on mild analgesics alone if they suffer from this degree of pain [10].

One of the most commonly used tools for pain assessment is the Brief Pain Inventory (BPI) which depends on several factors, including pain location, intensity, treatment, and effect on daily activities [9-11]. The scale ranges from 0 to 10 where 0 implies that there is no pain and 10 implies that the pain is as bad as you can imagine [9,11,12].

Despite published guidelines and education on the assessment and management of cancer-related pain, underestimated or undertreated pain continues to be a considerable worldwide public health concern in patients with solid and hematological malignancies; moreover, the adequate and usual self-reporting assessment of pain is the first step for effective and personalized treatment [4]. Consequently, inadequate pain assessment prevents optimal treatment among cancer patients [8]. This research aims to assess physicians’ adherence to WHO guidelines in the management and assessment of pain in oncology patients based on the available pain score in the Princess Noorah Oncology Center (PNOC) at the King Abdulaziz Medical City in Jeddah (KAMC-JD).

## Materials and methods

This cross-sectional, retrospective study investigated physicians’ adherence to WHO guidelines in the assessment and management of pain among oncology patients based on the available pain score in the PNOC at the KAMC-JD. KAMC-JD is a tertiary hospital with 558 functional beds located within the Makkah region (western region) of the Kingdom of Saudi Arabia. The PNOC in KAMC-JD is a tertiary cancer center with an 88-bed adult general oncology inpatient unit, a 22-bed bone marrow transplant unit, and a 32-bed pediatric hematology and oncology unit. The study design was approved by the King Abdullah International Medical Research Center ethics review board (SP21J/216/05), and the informed consent was waived because of the study design. The study was conducted from January 1, 2020, till December 31, 2020. We included all adult cancer patients who were admitted to the PNOC during the study period and excluded patients who did not fit the inclusion criteria or requested privacy. A total of 2,133 patients fit the inclusion criteria. We calculated the sample size using Raosoft software with a confidence level of 95% and an error margin of 5%, resulting in 326 patients. However, we included a total of 451 patients through computer-generated random sampling. Data were extracted from the BESTCare Health Information System (HIS) and our variables included pain, assessment scoring, and opioid prescribed (drug name and dosage per day). Demographic data including age, gender, area of residence, code status, co-morbidities, admitting diagnosis, the specialty the patient was admitted under, date and time of admission, the length of stay in each admission, and previous opioid usage (drug name and dosage per day) were collected.

The opioid dose was expressed as morphine equivalent daily dose (MEDD) using the standardized factors, as reported previously [13,14]. Based on the WHO’s analgesics ladder, opioids were classified into weak opioids (Tylenol, codeine, and tramadol) and potent opioids (morphine, oxycodone, hydromorphone, fentanyl, and methadone) [15]. Non-opioid analgesics included acetaminophen and NSAIDs, as well as adjuvant analgesics such as pregabalin, gabapentin, and venlafaxine.

Statistical analysis

Descriptive statistics were used to express categorical variables (frequencies and percentages) and numerical variables (means and standard deviation [SD] or median and interquartile range [IQR] as appropriate). Categories of pain intensity were defined as mild (0-3), moderate (4-6), and severe (7-10). For the univariate analysis, differences in the pain intensity based on drug prescription and the prescription of weak/strong opioids were tested using Pearson’s chi-square test for categorical variables, whereas the association between MEDD and pain intensity was assessed using Kruskal-Wallis one-way analysis of variance. In the multivariate analysis, the predictors of drug prescription were assessed using a logistic regression analysis (using the Enter method), where pain intensity was the independent variable. The analysis was adjusted for the following demographic and clinical characteristics: age, gender, marital status, body mass index (BMI), previous use of opioids, and the laboratory parameters assessed on admission. Results were reported as odds ratios (ORs) and their respective 95% confidence intervals (95%CIs). Statistical analysis was performed using SPSS version 26 (IBM Corp., Armonk, NY). Statistical significance was considered at p < 0.05.

## Results

Demographic, clinical, and admission-related characteristics

The data of 451 patients with cancer were reviewed between January 1 and December 31, 2020. The mean ± SD age of the patients was 55.3 ± 15.3 years, and the mean ± BMI was 26.2 ± 7.3 kg/m^2^ (Table 1). More than half of the patients were females (53.7%) and married (89.1%). Details about baseline clinical characteristics and the laboratory parameters are listed in Table 1.

**Table 1:** The demographic and clinical characteristics of patients at baseline (n = 451). *Descriptive statistics for variables with missing data, including the heart rate (n = 1) and albumin (n = 17). BMI: body mass index; SD: standard deviation

Parameter	Category	Frequency/Mean	Percentage/SD
Age, years	Mean and SD	55.25	15.33
Height, cm	Mean and SD	159.61	16.98
Weight, kg	Mean and SD	68.64	33.95
BMI, kg/m^2^	Mean and SD	26.16	7.25
Gender	Male	209	46.3
	Female	242	53.7
Marital status	Single	37	8.2
	Married	402	89.1
	Divorced	4	0.9
	Widow	8	1.8
Coding status	Full code	419	92.9
	No code	32	7.1
Body temperature, °C	Mean and SD	36.90	0.41
Respiratory rate	Mean and SD	20.26	2.48
Heart rate*	Mean and SD	88.94	17.32
Systolic blood pressure, mmHg	Mean and SD	124.03	20.96
Diastolic blood pressure, mmHg	Mean and SD	71.00	14.45
Albumin*	Mean and SD	37.15	5.60
Hemoglobin	Mean and SD	12.91	11.03

**Figure 1:**
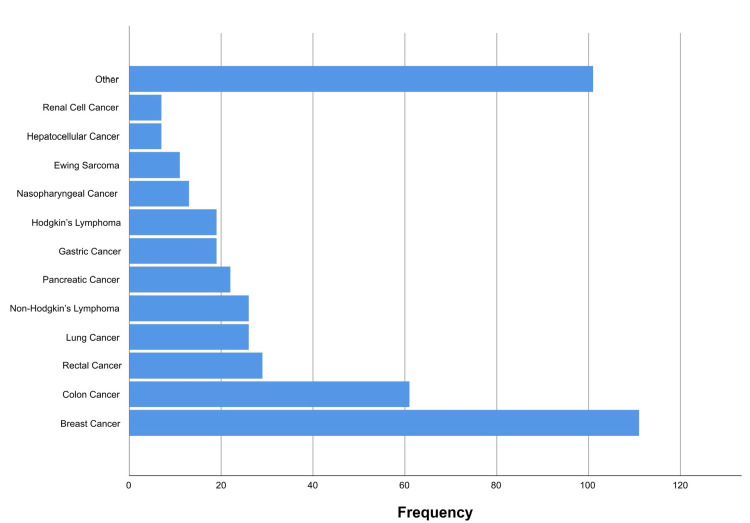
Patients’ type of cancer.

Regarding hospitalization characteristics, data of diagnosis on admission was available for 449 (99.6%) patients. The majority of patients (n = 447, 99.6%) were admitted with one type of cancer, one (0.2%) patient with two types of cancer (cancer of the ovary and gallbladder), and another with three types of cancer (cancer of the breast and ovary and appendix adenocarcinoma). Breast cancer was the most common malignancy (n = 111, 24.6%) followed by colon cancer (13.5%) and rectal cancer (6.4%) (Figure 1).

**Figure 2:**
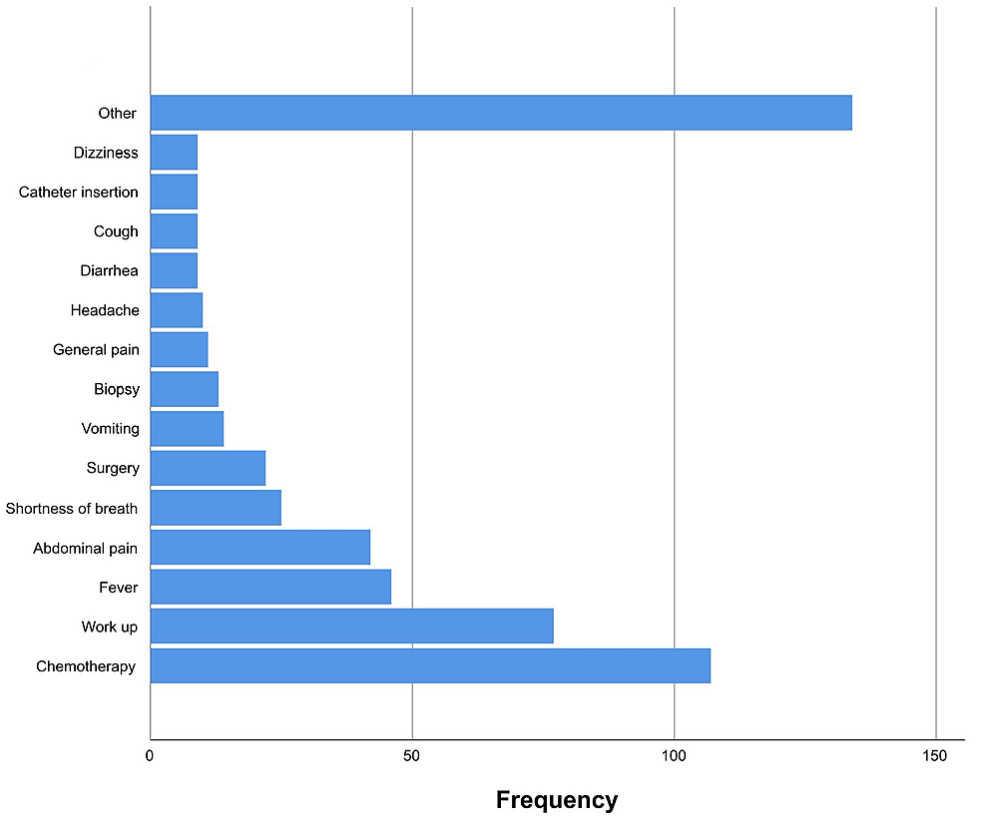
Patients’ admission reason.

Regarding the reasons for admission, 537 causes were retrieved from the records of 445 patients (representing 98.7% of the sample). The most common reason for hospitalization was undergoing chemotherapy (19.9%), workup (14.3%), and fever (8.6%) (Figure 2).

**Figure 3:**
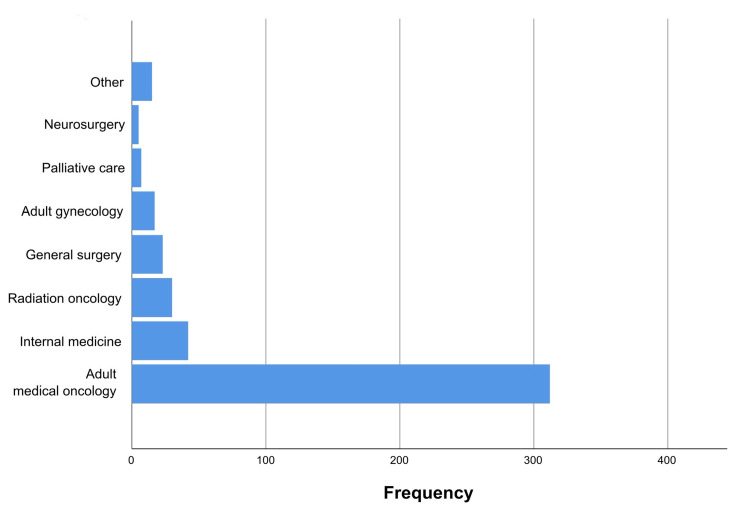
Patients’ admitting service.

The majority of patients were admitted to the department of adult medical oncology (69.2%) and internal medicine (9.3%) (Figure 3), and the mean length of hospital stay was 7.1 ± 6.1 days.

Characteristics of pain assessment

The pain was assessed in almost all patients (n = 450, 99.8%). It was categorized as mild in 386 (85.6%) patients, moderate in 46 (10.2%) patients, and severe in 19 (4.2%) patients.

Patterns of opioid use and opioid prescription

In general, 95 (21.1%) patients reported previous use of an opioid medication with a median (IQR) MEDD of 30 mg (20-45 mg). After the initial pain assessment, 218 opioid medications were prescribed for 203 (45.0%) patients. A total of 188 (41.7%) patients received one medication and 15 (3.3%) patients received two medications. In addition, 95 (21.1%) patients received weak opioids and 123 (27.3%) patients received potent opioids. Tramadol and morphine were the most commonly prescribed weak and strong opioids, respectively. The median (IQR) MEDD of the prescribed opioids was 40 mg (30-69 mg).

Relationship between opioid prescription and pain intensity at initial assessment

In the univariate analysis, opioid prescription was significantly higher among patients with moderate (76.1%) and severe pain (89.5%) compared to those with mild pain (39.1%; p < 0.0001) (Table 2). Based on the multivariate logistic regression analysis controlled for demographic and clinical characteristics, physicians were more likely to prescribe opioids for patients with moderate (OR = 5.85, 95% CI = 2.67 to 12.84; p < 0.0001) and severe pain (OR = 16.01, 95% CI = 3.39 to 75.58; p < 0.0001), considering mild pain as a reference category. However, there were no significant differences between the categories of pain intensity in terms of the opioid class (weak or strong opioids) and the prescribed MEDD (Table 2).

**Table 2:** The relationship between patterns of opioid prescription and pain intensity. IQR: interquartile range; MEDD: morphine equivalent daily dose

Parameter	Category	Pain intensity			P-value
		Mild	Moderate	Severe	
Opioids prescription, n (%)	No	235 (60.9)	11 (23.9)	2 (10.5)	<0.0001
	Yes	151 (39.1)	35 (76.1)	17 (89.5)	
Opioids class, n (%)	Weak only	63 (41.7)	13 (37.1)	4 (23.5)	0.651
	Strong only	78 (51.7)	19 (54.3)	11 (64.7)	
	Both weak and strong	10 (6.6)	3 (8.6)	2 (11.8)	
MEDD, median (IQR)	mg	40 (20–60)	36 (30–40)	36 (24–90)	0.670

Patterns of prescription of analgesics

**Figure 4:**
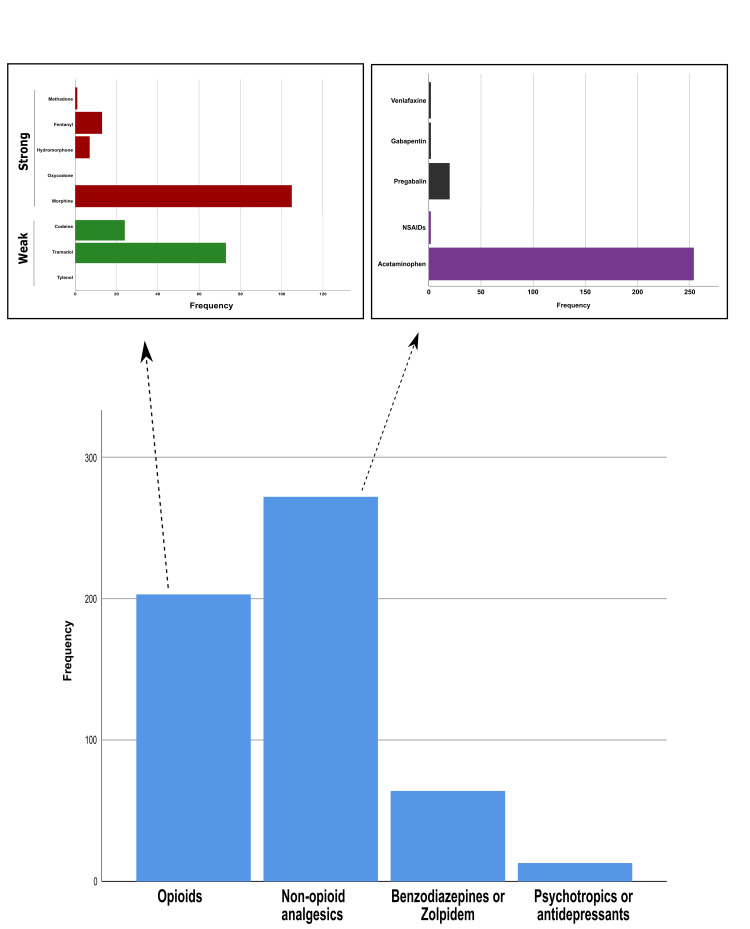
Pattern of opioid use and other analgesic prescription.

Physicians provided prescriptions for 280 non-opioid analgesics to 272 (60.3%) patients. Acetaminophen was the most commonly prescribed medication (n = 254, 90.7%), and pregabalin was the most frequently prescribed adjuvant (n = 20, 7.1%) (Figure 4). Analgesics were prescribed in a combination with opioids in 139 (30.8%) patients.

Relationship between analgesic prescription and pain intensity at initial assessment

The analysis of analgesic prescriptions showed that patients with moderate or severe pain (84.8% and 89.5%, respectively) received at least one analgesic medication more frequently compared to those with mild pain (53.9%; p < 0.0001) (Table 3). Moreover, physicians prescribed acetaminophen or NSAIDs more frequently to patients with moderate or severe pain (80.4% and 84.2%, respectively) than patients with mild pain (52.6%; p < 0.0001). However, pain intensity did not influence the prescription of adjuvant analgesics (Table 3).

**Table 3:** The relationship between patterns of analgesic prescription and pain intensity. NSAIDs: non-steroidal anti-inflammatory drugs

Parameter	Category	Pain category			P-value
		Mild	Moderate	Severe	
Number of prescribed analgesics (all)	0	170 (44)	7 (15.2)	2 (10.5)	<0.0001
	1	208 (53.9)	39 (84.8)	17 (89.5)	
	2	8 (2.1)	0 (0)	0 (0)	
Number of prescribed NSAIDs or acetaminophen	0	183 (47.4)	9 (19.6)	3 (15.8)	<0.0001
	1	203 (52.6)	37 (80.4)	16 (84.2)	
Number of prescribed adjuvant analgesics	0	365 (94.6)	44 (95.7)	18 (94.7)	0.952
	1	21 (5.4)	2 (4.3)	1 (5.3)	

As per the adjusted logistic regression analyses, physicians were more likely to prescribe at least one analgesic to patients with moderate (OR = 5.73, 95% CI = 2.17 to 15.11; p < 0.0001) and severe pain (OR = 4.81, 95% CI = 1.06 to 21.75; p = 0.041) regardless of their demographic and clinical characteristics. The independent association remained consistent for analgesic prescriptions for those with moderate pain only with the adjustment for opioid prescription (OR = 4.9, 95% CI = 1.83 to 13.08; p = 0.002). Furthermore, the prescription of NSAIDs or acetaminophen was independently associated with moderate pain intensity in the models adjusted for clinical and demographic characteristics (OR = 4.63, 95% CI = 1.97 to 10.84; p < 0.0001) and opioid prescription (OR = 4.08, 95% CI = 1.72 to 9.69; p = 0.001 (Table 4). Interestingly, pain intensity was independently associated with the combined prescription of an opioid drug with either acetaminophen or NSAIDs (OR = 7.54, 95% CI = 3.72 to 15.28; p < 0.0001 for moderate pain and OR = 9.07, 95% CI = 2.98 to 27.63; p < 0.0001 for severe pain) (Table 4).

**Table 4:** Regression analysis of the relationship between pain intensity at initial assessment and the prescription of analgesics. ¥Model 1: results were adjusted for demographic and clinical characteristics. ¶Model 2: results were adjusted for demographic and clinical characteristics, as well as opioid prescription. NA: non-applicable due to the inclusion of opioids in the analysis. NSAIDs: non-steroidal anti-inflammatory drugs

Parameter	Category	Model 1^¥^		Model 2^¶^	
		OR (95% CI)	P-value	OR (95% CI)	P-value
Prescription of ≥one analgesic (all types)	Mild pain	Reference		Reference	
	Moderate pain	5.73 (2.17 to 15.11)	<0.0001	4.9 (1.83 to 13.08)	0.002
	Severe pain	4.81 (1.06 to 21.75)	0.041	3.84 (0.83 to 17.72)	0.084
Prescription of NSAIDs or acetaminophen	Mild pain	Reference		Reference	
	Moderate pain	4.63 (1.97 to 10.84)	<0.0001	4.08 (1.72 to 9.69)	0.001
	Severe pain	3.43 (0.95 to 12.42)	0.06	2.88 (0.78 to 10.64)	0.112
Combined prescription of opioids plus NSAIDs or acetaminophen	Mild pain	Reference		NA	
	Moderate pain	7.54 (3.72 to 15.28)	<0.0001	NA	
	Severe pain	9.07 (2.98 to 27.63)	<0.0001	NA	

Patterns of prescription of benzodiazepines and psychotropics

In the present study, a total of 68 benzodiazepines (BZD) or zolpidem (BZD/zolpidem) were prescribed for 64 patients (representing 14.2% of the entire sample). Lorazepam (38.2%) and midazolam (30.9%) were the most frequently prescribed BZDs. Other characteristics of the prescribed BZD/zolpidem are listed in Table 5.

**Table 5:** Patterns of prescription of benzodiazepines or zolpidem. Data were available for 64 patients (receiving 68 benzodiazepines/zolpidem). *The percentage value was based on the entire sample. IV: intravenous; IQR: interquartile range

Parameter	Category	N	%
Number of prescribed medications*	1	60	13.30
	2	4	0.89
Drug name	Midazolam	21	30.88
	Zolpidem	16	23.53
	Lorazepam	26	38.24
	Clonazepam	2	2.94
	Diazepam	2	2.94
	Oxcarbazepine	1	1.47
Route of administration	IV	24	35.29
	Oral	41	60.29
	Subcutaneous	3	4.41
Dose, mg	Median (IQR)	2	1–5

Regarding psychotropics and antidepressants, 14 medications were prescribed for 13 (2.9%) patients. Physicians used mirtazapine (28.6%) and olanzapine (21.4%) more frequently, and the majority of these medications were given orally (85.7%). Details about the patterns of psychotropic/antidepressant prescriptions are listed in Table 6.

**Table 6:** Patterns of prescription of psychotropics/antidepressants. Data were available for 13 patients (receiving 14 psychotropics/antidepressants). *The percentage value was based on the entire sample. IQR: interquartile range

Parameter	Category	N	%
Number of prescribed medications*	1	12	2.66
	2	1	0.22
Drug name	Olanzapine	3	21.43
	Mirtazapine	4	28.57
	Citalopram	2	14.29
	Chlorpromazine	1	7.14
	Escitalopram	1	7.14
	Venlafaxine	2	14.29
	Quetiapine	1	7.14
Route of administration	Oral	12	85.71
	Subcutaneous	1	7.14
	Missing	1	7.14
Dose, mg	Median (IQR)	20	8.75–35

## Discussion

Pain management is an integral part of oncology care to optimize the quality of life and survival of patients [16]. Inadequate knowledge and malpractice of pain management are well-cited clinician-related barriers in the palliative care niche [9]. Therefore, it is necessary to evaluate the current practice of cancer pain management by evaluating the practice of pain assessment and the patterns of analgesic prescriptions. In this study, we found that pain intensity was a significant predictor of opioid prescription regardless of the demographic and clinical characteristics of patients at baseline. Furthermore, non-opioid analgesics (acetaminophen and NSAIDs) were more likely to be prescribed to oncology patients with moderate and severe intensity compared to patients with mild pain.

This study revealed that pain assessment was performed and recorded in the electronic records system for the vast majority of patients (99.8%). This is in compliance with the guidelines of cancer pain management [8], which necessitates conducting a comprehensive pain assessment consistent with the patient’s comfort along with undertaking a detailed history and physical examination at the initial presentation. In the United States, El Rahi et al. conducted a chart review of 99 patients with solid tumors [9]. They found that pain scores were documented for 84% of the included patients over a six-month follow-up period. This was comparable to a proportion of 81% of pain documentation in an early review of cancer patients who were hospitalized in U.S. cancer centers [17]. Pain documentation is an important element of the palliative care service, and pain reporting for approximately all patients in our study is primarily attributable to the high compliance of providers at a tertiary hospital in Jeddah, Saudi Arabia.

Additionally, the fact that the pain score was documented for a proportion of patients who had a history of opioid use indicates that pain re-assessment is effectively performed in our setting. This also reflects the high-quality practice of assessment and re-assessment to monitor the performance of medications and to ensure that the prescribed treatments are appropriate and safe. However, data regarding pain reassessment shortly after opioid administration was not available because these were not collected from patient records. Pain reassessment rates were generally low in other studies in the literature (ranging from 44% to 61%) [18,19]. Indeed, inappropriate re-assessment may cause a significant delay in pain relief as well as a delay in transitioning to oral opioids, as indicated in the guidelines of the National Comprehensive Cancer Network [20]. Therefore, future retrospective studies may consider this parameter to investigate physicians’ practice regarding pain reassessment and the subsequent opioid dose titration.

The present study showed that physicians had adhered to the published WHO guidelines [8]. The patterns of drug prescription, including non-opioid analgesics, opioids, and adjuvants showed that the physicians have followed the WHO three-step ladder of cancer pain management. In essence, combination therapy of mild analgesics with an opioid was more likely to be prescribed for patients with moderate pain, and the association was stronger for those with severe pain. Such a pattern was also in agreement with the recommended combination of paracetamol and/or NSAIDs with an oral opioid in the WHO Model List of Essential Medicines in palliative care [21].

The current study has also shown that opioids were prescribed according to the published guidelines, where the odds of opioids prescription were significantly high with increased pain intensity. Nevertheless, we could not find significant differences in the prescription of weak and strong opioids as well as the MEDD dose across different pain intensities. Presumably, the lack of significant differences may be due to the small sample size of patients who received opioids (a dedicated power analysis was not performed for such a subset of patients). Another possible explanation is that the practice of optimal opioid selection was not adequate among physicians. Accordingly, palliative care educational programs should be meticulously tailored based on the optimal use of different opioids along with their recommended dosages.

In this study, acetaminophen (paracetamol) was the most commonly used non-opioid analgesic. Although current WHO guidelines and the classic analgesics ladder recommend the use of paracetamol in mild-to-moderate pain, patients with higher intensities of pain who are already being treated with a potent opioid may be less likely to gain any additional benefit [16,20,22-23]. In addition, long-term data from randomized controlled studies are not available to provide high-quality evidence, with no superiority of paracetamol over NSAIDs [24]. The latter class of medications has also induced favorable benefits over placebo for cancer pain, but the available evidence was limited by the heterogeneity among different trials [25]. Overall, the choice of the suitable analgesics does not follow a distinct clinical recommendation; yet, it should be based on individualized therapeutic planning according to the assessment of each patient, the site and type of pain, and the optimal dose that achieves effective analgesia.

In this study, pain assessment was performed by one of the most commonly used tools BPI which concisely evaluates the location and intensity of pain, and it helps reveal the impact of pain on daily life activities [26]. In fact, the BPI showed higher validity and reliability measures than the verbal rating scale among cancer patients, and it was also validated among Arab populations [27,28]. However, the study may be limited by the retrospective nature of data collection; such a limitation may be resolved by conducting a prospective evaluation of prescription patterns to reveal reliable causal relationships between drug prescription and pain intensity. Furthermore, we could not assess the patterns of drug prescription according to the grade of cancer. Finally, we could not assess the patterns of opioid use by patients, which has been considered an essential element to reduce the risk of misuse and underuse during the treatment period [29,30]. Opioids costs may be another important factor in the patterns of opioids use and prescription patterns. Consequently, the economic and personal factors that may influence opioid use should be investigated.

## Conclusions

The practice of pain documentation for patients with cancer was adequate at the KAMC-JD as indicated by reporting the pain scores of 99.8% of inpatients. Patients with moderate and severe pain were more likely to receive opioids and a combination of opioids plus non-opioid analgesics, whereas the prescription of analgesics was predicted by moderate cancer pain. Relevant educational programs in palliative care should be taught to physicians to guide opioid dosing and the prescription of weak or strong opioids. Future prospective studies should consider the patterns of drug prescription based on the type and stage of cancer and the economic and patient-related patterns of opioids use, as well as the temporal changes in analgesic dosing on admission and throughout the hospitalization period.
